# Metagenome-assembled complete genome of *Bohxovirus*, a virulent bacteriophage involved in the prediction of hospital-acquired pneumonia in intubated critically ill patients

**DOI:** 10.1128/mra.00592-25

**Published:** 2025-10-29

**Authors:** Hussein Anani, Grégory Destras, Hadrien Regue, Simon Bulteau, Céline Bressollette-Bodin, Antoine Roquilly, Laurence Josset

**Affiliations:** 1 GenEPII sequencing platform, Institut des Agents Infectieux, Hospices Civils de Lyon, Lyon, France; 2 CIRI, Centre International de Recherche en Infectiologie, Team VirPath, Univ Lyon, Inserm, U1111, Université Claude Bernard Lyon 1, CNRS, UMR5308, ENS de Lyon, Lyon, France; 3 Laboratoire de Virologie, Institut des Agents Infectieux, Laboratoire associé au Centre National de Référence des virus des infections respiratoires, Hospices Civils de Lyon, Lyon, France; 4 Nantes Université, Center for Research in Transplantation and Translational Immunology, UMR 1064 27045, Nantes, France; 5 Nantes Université, CHU Nantes, Service de virologie 27045, Nantes, France; 6 Nantes Université, CHU Nantes, Service d’anesthésie réanimation 27045, Nantes, France; 7 Department of Microbiology and Immunology, University of Melbourne, Peter Doherty Institute for Infection and Immunity 2281, Melbourne, Victoria, Australia; Loyola University Chicago, Chicago, Illinois, USA

**Keywords:** bacteriophage genome, respiratory virome, viral metagenomics

## Abstract

We present the complete genome of a *Bohxovirus* species, a virulent phage targeting *Prevotella jejuni*, reconstructed from viral metagenomes in respiratory endotracheal aspirates of intubated critically ill patients. The 98-kbp bacteriophage, belonging to the *Suoliviridae* family, does not contain genes associated with antibiotic resistance or bacterial virulence.

**CLINICAL TRIALS:**

ClinicalTrials.gov numbers: NCT02003196 and NCT04793568.

## ANNOUNCEMENT

Hospital-acquired pneumonia (HAP) is the most common nosocomial infection in intensive care unit (ICU) patients, leading to increased mortality (1). In a recent study, we characterized the endotracheal virome of ICU patients and revealed that respiratory virome was dominated by bacteriophages (2, 3). We identified a viral signature classifying patients into upcoming HAP or no HAP. In the previous study (3), we sampled 121 endotracheal aspirates from 87 patients to study the role of the respiratory virome in HAP pathogenesis.

Total nucleic acids were extracted from samples using the Qiagen EZ1 Advanced XL extractor and amplified with the WTA2 Kit, followed by purification with QIAquick spin columns. Libraries were prepared using the Nextera-XT Kit and sequenced on NovaSeq-6000 with 2 × 100 bp paired-end reads. Raw data downloaded from the BioProject PRJNA1132989, were processed using viral_metagenomics_pipeline v1.0.0 (https://github.com/genepii/HAP2-IBIS-Virome). Human reads were removed with SRAHumanScrubber v2.2.1 (default parameters) (https://github.com/ncbi/sra-human-scrubber), followed by quality trimming using Cutadapt (4) v4.6 (default parameters). Taxonomic classification was performed with Kraken2 (5) v2.1.2 (k-mer-based assignment; min-hit-groups = 2, confidence = 0.1). Viral contigs from each sample were assembled individually using SPAdes (6) v.3.14.0 with the --meta parameter, dereplicated with cd-hit (7) v4.7, and quality checked with Checkv (8) v1.1.3, using the default parameters. Viral metagenomic sequencing yielded a total of 276 gigabases (Gb) of raw data for which 61 Gb were assigned as viral reads.

Among the 66 viral and conserved operational taxonomical units (vcOTUs) reported in the HAP signature, the longest (98 kbp) vcOTU was a *Caudoviricetes* bacteriophage (vcOTU56 as reported in Anani and colleagues [3]). vcOTU56 was analyzed using Phabox2 v2.1.12 (9) with default parameters, which integrates several modules: Phatyp predicts whether a virus is virulent or temperate by analyzing genomic features and protein content. PhagCN predicts potential viral hosts by leveraging sequence similarity, k-mer patterns, and co-occurrence networks to identify bacterial hosts. Phamer performs gene-level annotation and clustering by comparing viral proteins against the most recent International Committee on Taxonomy of Viruses database release to assign taxonomic information. vcOTU56 was annotated as a *Bohxovirus*, classified within the *Suoliviridae* family, has a virulent lifestyle, and infects *Prevotella jejuni*, a bacterium that inhabits the human oral and gut microbiomes (10).

**Fig 1:**
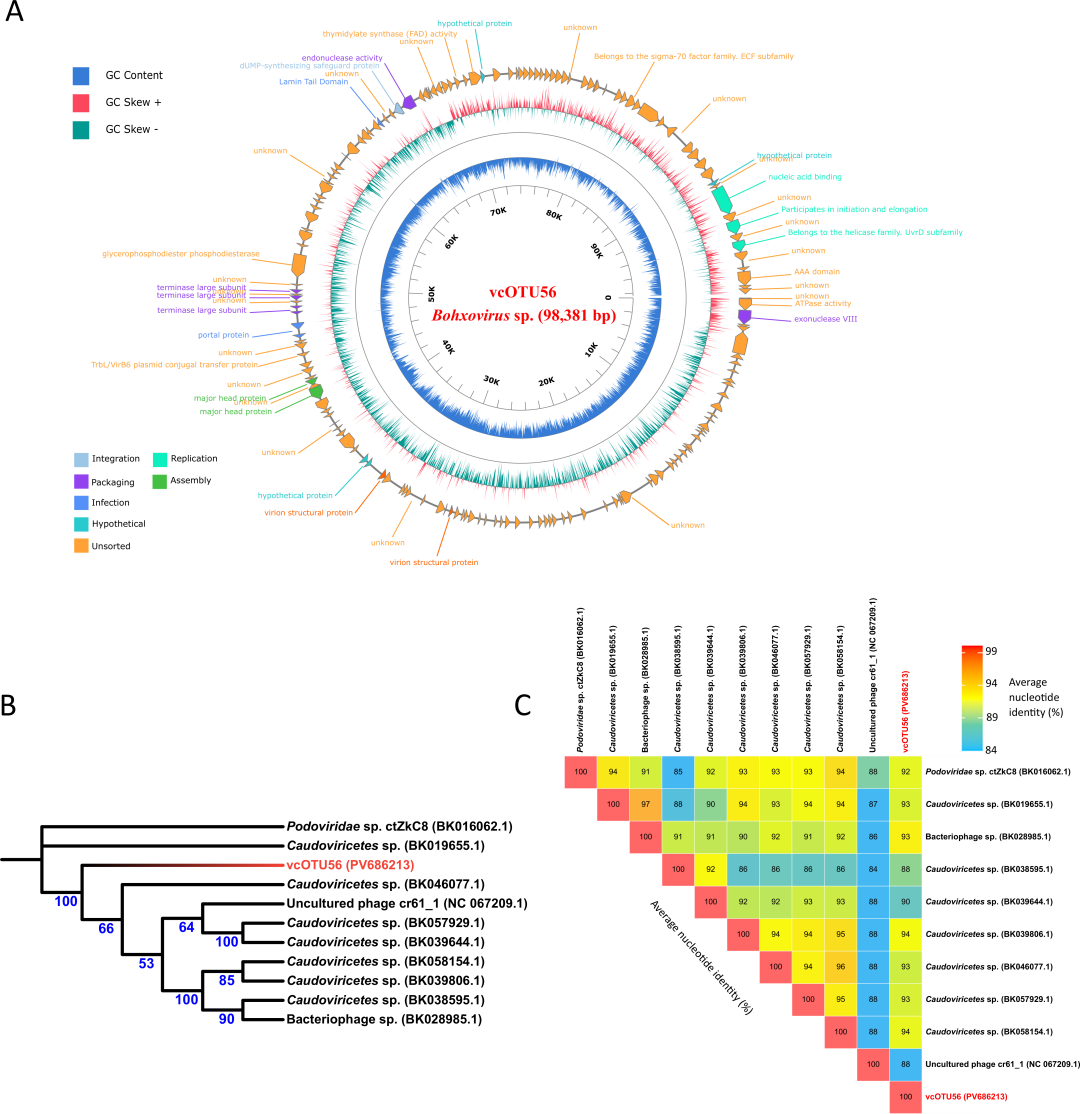
Genomic and comparative analysis of the vcOTU56. (**A**) A graphical circular map of the vcOTU56 genome. From the outside to the center: viral protein-coding genes. G + C skew and G + C content. (**B**) Maximum-likelihood phylogenetic tree showing the position of vcOTU56 (in red) relative to other closely related viral taxa. Sequences were aligned using MAFFT v7.508 (13) and trimmed with trimAl v1.4.1 (14), and the tree was constructed with IQ-TREE2 v2.2.2.3 using 1,000 rapid bootstrap replicates (15). Node bootstrap values are highlighted in blue. (**C**) Matrix showing the average nucleotide identity (ANI) values between vcOTU56 and other closely related taxa.

vcOTU56, present in 29% of samples, with reads remapping (mean relative abundance of 0.05%), has a complete circular genome of 98,381 bp with a sequencing depth of 2,372× and GC content of 33.06% (100% completeness and 0% contamination). Genome annotation using Prokka v1.14.6 (11) with the --kingdom Viruses parameter predicts 168 viral genes and 5 tRNA genes. vcOTU56 genome was visualized using PhageScope (12) (Fig. 1A). Phylogenetic analysis of vcOTU56 and the 10 closest BLASTn-identified sequences from nucleotide-NCBI database showed that vcOTU56 formed a separate group (Fig. 1B). Interestingly, all closest sequences, like vcOTU56, were predicted to belong to *Bohxovirus* (Table 1).

**TABLE 1:** Genome features of vcOTU56 closely relative sequences

Closest relative sequence by BLASTn	Phabox2 classification	Phabox2 confidence	CheckV quality	Max score	Total score	Query cover	E value	Identity (%)	Length (bp)	Accession
MAG TPA_asm: *Caudoviricetes* sp. isolate ctRU85	*Bohxovirus*	High confidence	Complete	29,990	1.37E + 05	95%	0	92.7	99,611	BK019655.1
MAG TPA_asm: *Podoviridae* sp. ctZkC8	*Bohxovirus*	High confidence	Complete	28,692	97,088	70%	0	91.94	93,668	BK016062.1
MAG TPA_asm: *Bacteriophage* sp. isolate ctxN72	*Bohxovirus*	High confidence	Complete	27,366	1.32E + 05	95%	0	93.46	97,813	BK028985.1
Uncultured phage cr61_1	*Bohxovirus*	High confidence	Complete	23,893	61,579	59%	0	87.72	98,573	NC_067209.1
MAG TPA_asm: *Caudoviricetes* sp. isolate ctss16	*Bohxovirus*	High confidence	Complete	23,889	1.04E + 05	71%	0	92.6	100,941	BK046077.1
MAG TPA_asm: *Caudoviricetes* sp. isolate ctnLP2	*Bohxovirus*	High confidence	Complete	20,825	1.23E + 05	94%	0	89.64	102,343	BK039644.1
MAG TPA_asm: *Caudoviricetes* sp. isolate ctTb212	*Bohxovirus*	High confidence	Complete	20,040	31,089	21%	0	93.72	26,228	BK039806.1
MAG TPA_asm: *Caudoviricetes* sp. isolate ctJxI8	*Bohxovirus*	High confidence	Complete	18,454	69,215	45%	0	93.72	58,045	BK058154.1
MAG TPA_asm: *Caudoviricetes* sp. isolate ctR5o17	*Bohxovirus*	High confidence	Complete	17,998	46,892	34%	0	93.08	38,277	BK057929.1
MAG TPA_asm: *Caudoviricetes* sp. isolate ctdNw4	*Bohxovirus*	High confidence	Complete	17,860	1.02E + 05	86%	0	87.51	99,284	BK038595.1

vcOTU56 exhibits a 92.70% ANI with its closest sequence (*Caudoviricetes*_sp_isolate_ctRU85, Fig. 1C). In accordance with the 95% species demarcation threshold, these data suggest that vcOTU56 is a new species of the genus *Bohxovirus*, which warrants further investigation.

## Data Availability

The complete sequence of vcOTU56 has been deposited in GenBank under the accession number PV686213, and raw reads are available under the BioProject number PRJNA1132989.
